# Comparison of the effect between pioglitazone and metformin in treating patients with PCOS:a meta-analysis

**DOI:** 10.1007/s00404-017-4480-z

**Published:** 2017-08-02

**Authors:** Yifeng Xu, Yanxiang Wu, Qin Huang

**Affiliations:** 1 Department of Endocrinology, Changhai Hospital, the First Affiliated Hospital of the Second Military Medical University, Shanghai, People’s Republic of China; 2 Department of Endocrinology, The 463rd Hospital of PLA, Shenyang, People’s Republic of China

**Keywords:** PCOS, Pioglitazone, Metformin, Therapy

## Abstract

**Background:**

Pioglitazone was used to treat patients of PCOS in many researches, but the treatment has not been recognized by public or recommended by all the guidelines.

**Method:**

We conducted a meta-analysis of the related literatures to objectively evaluate the clinical effectiveness and safety by comparing pioglitazone with metformin administrated by PCOS patients. Searches were performed in Cochrane Library, EMBASE and PubMed (last updated December 2016).

**Results:**

Eleven studies among 486 related articles were identified through searches. Fixed effects and random effects models were used to calculate the overall risk estimates. The results of the meta-analysis suggest that improvement of the menstrual cycle and ovulation in pioglitazone treatment group was better than metformin group [OR = 2.31, 95% CI (1.37, 3.91), *P* < 0.001, *I* ^2^ = 41.8%]. Improvement of the F-G scores in metformin treatment group was better than pioglitazone group [SMD = 0.29, 95% CI (0.0, 0.59), *P* = 0.048, *I* ^2^ = 0.0%]. BMI was more elevated in pioglitazone group than in metformin group [SMD = 0.83, 95% CI (0.24, 1.41), *P* = 0.006, *I* ^2^ = 82.8%]. There were no significant differences of the other data between the two groups.

**Conclusions:**

This meta-analysis indicated that pioglitazone ameliorated menstrual cycle and ovulation better than metformin and metformin ameliorated BMI and F-G scores better than pioglitazone in treating patients with PCOS. Pioglitazone might be a good choice for the patients with PCOS who were intolerant or invalid to metformin for the treatment.

## Introduction

PCOS (polycystic ovary syndrome) is one of the most common endocrine disorders in women, and it affects about 5–7% women of reproductive age [1] who accounts for 30–60% of anovulatory infertility patients. Its basic characteristics are hyperandrogenism, chronic anovulation and polycystic ovaries. It increases the incidence of endometrial cancer and ovarian cancer. In addition, patients always have the character of insulin resistance, central obesity, impaired glucose, dyslipidemia, cardiovascular risk and subclinical atherosclerosis [2–4]. PCOS can increase the risk of type 2 diabetes in 5–10% [5–7], and 30–70% of patients with PCOS may be accompanied with obesity [8, 9]. The prevalence of IGT in patients with PCOS in America reached 30–35%, and 5% of these will develop to type 2 diabetes. Adolescent and adult women with PCOS usually need OGTT test of 75 g of glucose, and assessment of waist circumference, BMI, blood lipids, blood pressure and other metabolic factors are also needed. Metformin can ameliorate ovulation and metabolic risk of PCOS and it is currently used as second-line therapy in some PCOS guidelines [10]. Thiazolidinediones (TZDs) are a kind of highly selective synthetic agonists of PPAR-γ (peroxisome proliferation-activated receptor gamma) for the treatment of diabetes. TZDs, the classical insulin sensitizers include troglitazone, rosiglitazone and pioglitazone. The pathogenesis of PCOS may be related to the alteration of PPAR-γ gene, and PPAR-γ seems to play an important role in fertility and metabolism through the effects of its different hypotypes. For example, PPAR-γ1 can specifically regulate ovarian function [11]. Insulin resistance is an important aspect of PCOS and has been observed not only in obese but also in lean women with PCOS and seems to be an intrinsic part of the syndrome [12, 13]; PPAR-γ agonists can decrease androgen synthesis in ovaries by ameliorating peripheral insulin resistance indirectly [14]. Thus, those provide theoretical and practical bases for the treatment of PCOS with TZDs therapy. The treatment of PCOS with TZDs has been investigated in many animals and clinical studies and trials, and most of the trials showed an effective therapeutic result. But it still lacks sufficient evidence-based supports. There are no large-scale clinical trials to verify the efficacy and safety of TZDs drugs for PCOS [15]. So the treatment has not been recognized by public or recommended by all the guidelines. We conducted a meta-analysis of the related literatures to objectively evaluate the clinical effectiveness and safety by comparing pioglitazone with metformin administrated by PCOS patients.

## Materials and methods

### Literature search

We searched the literatures on Cochrane Library, EMBASE and PubMed with an end date of December 2016. The last searching time was on January 10, 2017. The search terms in full text included the following: “pioglitazone”, “metformin”, “polycystic ovary syndrome” or “PCOS”, and “randomized controlled trial” or “randomized”. Publication time, genre and languages were not limited.

### Outcome measures

The main outcome was to compare the therapeutic effect between pioglitazone and metformin prescribed for the patients with PCOS. Therapeutic parameters included menstrual cycle, body mass index (BMI), waist hip ratio (WHR), waist circumference (WC), acne, F-G score for hirsutism, fasting blood sugar (FBS), insulin (INS), homeostasis model of assessment for insulin resistance index (HOMA-IR), total cholesterol (TC), triglyceride (TG), free testosterone (T), dehydroepiandrosterone (DHEA), free androgen index (FAI), luteinizing hormone/follicle-stimulating hormone (LH/FSH), sex hormone binding globulin (SHBG), aspartate amino transferase (AST) and glutamic pyruvic transaminase (ALT).

### Selection criteria

Identified studies were included in the meta-analysis if [1] the patients were diagnosed with PCOS definitely and the diagnostic criteria conformed to the Rotterdam criteria of the European Society of Human Reproduction and Embryology (ESHRE) and American Society for Reproductive Medicine (ASRM) in 2003. [2] The randomized controlled study (RCT) was conducted in the literature. [3] Groups included at least pioglitazone therapy group and metformin therapy group, and there was comparison between the two groups. [4] Outcome measures included at least one of the above therapeutic parameters in the study. Studies were excluded if [1] the therapeutic group combined using other agents with pioglitazone or metformin. [2] Test design was not reasonable or lacked of effective control. [3] Review, case report, or animal experiments would be excluded.

### Data extraction and statistical analysis

No patient consent or ethical approval was required because analyses were based on previously published studies. The first two authors of this article have collected the literatures according to the criteria; the two authors, respectively, evaluated the quality of the literatures independently and negotiated to confirm [1] randomized method; [2] allocation concealment; [3] if patients, executors of the study, and surveyors were blinded; [4] patients withdrawal, loss to follow-up or dropped out. Different opinions of the first two authors of this article would be judged and concluded by the third author. Then the data of the literatures, including the first author’s name, year of publication, study period, average ages of the patients, therapeutic dose, therapeutic period, clinical effect and so on were extracted.

The software of StataSE 12.0 was used to analyze the data by meta-analysis and to draw forest plot. Inter-study heterogeneity among the trials was assessed using *Q* test. *I* ^2^ > 50% indicated significant heterogeneity and random effects model was used for statistical analysis. *I* ^2^ < 50% indicated no significant heterogeneity and fixed effects model was used for statistical analysis. The values of OR and 95% CI were used to describe the effect values of categorical data. Mean ± SD was used to measure the effect values of measurement data. Possible heterogeneity among the studies was investigated by meta-regression. Overall quality of the literatures was evaluated by sensitivity analysis. Publication bias was assessed by Begg’s and Egger’s analysis with Begg’s funnel plot. *P* < 0.05 was considered statistically significant.

## Results

### Literature search

**Fig. 1:**
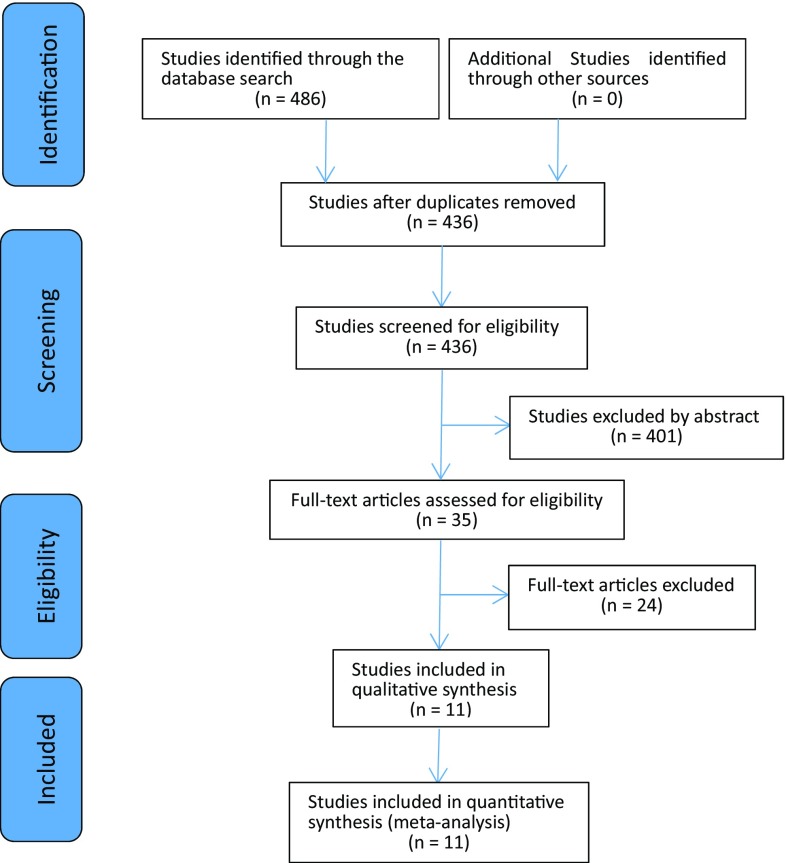
Search strategy for selection of trials. From: Lesley et al. [48]

Total 486 related articles were extracted from the three databases and 436 articles were left after removing duplication. Finally, 11 articles meeting the criteria [16–26] were left after the abstract and the full text had been read. Figure 1 shows the search strategy for selection of trials.

### Study characteristics

In total, there were 643 patients, of whom 319 patients were treated with pioglitazone and 324 patients treated with metformin. Table 1 shows the characteristics of the included randomized controlled trials. Detailed data are shown in Tables 2 and 3.

**Table 1:** Characteristics of the included randomized controlled trials

Authors	Publication year	Country	Randomized	Blind	Concealment	ITT	Baseline comparable	Cases P/M	Withdrawal P/M	Age (year) P/M	Time	Duration (month)	Dose P/M
Shahebrahimi	2016	Iran	Yes	NR	NR	NR	NR	28/28	NR	27.5 ± 3.68/27.57 ± 5.91	2012.5–2013.4	3	30 mg daily/500 mg 3 times daily
Chaudhry	2016	Pakistan	Yes	NR	NR	NR	NR	35/35	0	29.97 ± 5.28/30.37 ± 5.63	2015.6–2015.12	3	15 mg twice daily/500 mg 3 times daily
Sohrevardi	2016	Iran	Yes	No	NR	NR	Yes	28/28	7/6	27.52 ± 5.01/28.72 ± 6.31	2014.4–2015.5	3	30 mg daily/500 mg 3 times daily
Kashani	2013	Iran	Yes	Yes	Yes	Yes	Yes	25/25	5/5	21.2 ± 3.3/20.3 ± 4.6	2011.7–2012.5	1.5	15 mg twice daily/750 mg twice daily
Sangeeta	2012	India	Yes	Yes	NR	NR	Yes	50/50	8/7	18–30	NR	6	15 mg daily/500 mg twice daily
Ziaee	2012	Iran	Yes	NR	NR	NR	Yes	26/26	0	25.23 ± 4.52/25.32 ± 4.31	2008.4–2009.8	3	30 mg daily/500 mg 3 times daily
Navali	2012	Iran	Yes	NR	NR	NR	Yes	50/50	6/0	28.8 ± 5.2/26.8 ± 4.5	2008–2009	6	15 mg twice daily/500 mg 3 times daily
Naka	2011	Greece	Yes	NR	NR	NR	Yes	15/15	1/0	23.6 ± 5.1/22.2 ± 3.6	NR	6	30 mg daily/850 mg twice daily
Cho	2009	UK	Yes	NR	NR	NR	Yes	10/10	0	26.4 ± 1.5	NR	3	45 mg daily/500 mg 3 times daily
Ortega	2005	México	Yes	NR	NR	NR	Yes	27/30	10/13	28.8 ± 0.9/28.6 ± 0.7	NR	6	30 mg daily/850 mg 3 times daily
Ortega	2004	México	Yes	NR	NR	NR	Yes	25/27	5/6	28.8 ± 0.9/29 ± 0.8	NR	6	30 mg daily/850 mg 3 times daily

*ITT* intention-to-treat (analysis), *NR* not reported, *P/M* pioglitazone group/metformin group

**Table 2:** The difference value between pre and after treatment

Authors	Group	FBS (mg/dL)		INS (μ/L)		HOMA-IR		BMI		WHR		Ovulation	F-G score	
		Mean	SD	Mean	SD	Mean	SD	Mean	SD	Mean	SD		Mean	SD
Shahebrahimi (2016)	P	−4.21	8.64	−6.6	44.23			0.27	4.42			15/28		
	M	−3.86	10.89	−10.57	11.47			−0.28	4.41			15/28		
Chaudhry (2016)	P											29/35		
	M											19/35		
Sohrevardi (2016)	P	−9	9	−3.8	6.3	−1	1.4	0.6	4.7	0.01	0.055	10/15		
	M	−5.4	4.7	−6.8	9.36	−1.9	2.34	−0.1	4.06	0	0.034	5/19		
Kashani (2013)	P	−4.3	19.92	−3.31	8.2	−0.16	0.85	0.57	3.72	0.02	0.072			
	M	−4.53	20.77	−3.07	8.66	−0.14	0.94	−0.97	3.34	−0.02	0.07			
Sangeeta (2012)	P			−21.1	7.62	−8.19	5.11						−6.43	5.4
	M			−6.95	6.71	−4.01	3.61						−8.08	5.25
Ziaee (2012)	P	−7.04	10.3	−5.72	6.66	−28.89	36.36	0.01	2.58					
	M	−8.2	10.93	−5.99	8.65	−31.33	43.48	−0.62	2.93					
Navali (2012)	P											22/32		
	M											13/31		
Naka (2011)	P	2	6.56	−4.6	5.21			1.3	5.56	−0.01	0.056		−1.6	2
	M	−3	5.57	−2.8	4.99			−0.1	6.5	0.02	0.056		−1.2	2.55
Cho (2009)	P			−8	2.51	−2	0.69	1.1	1.8					
	M			−1.7	2.65	−0.5	0.56	−1.1	1.85					
Ortega (2005)	P	−1.7	2.36	−19.1	1.57	−4.34	0.37	1.7	1.15	−0.02	0.02	14/17	−5	0.78
	M	−3.7	2.78	−12.2	3.49	−2.9	0.87	−2.1	1.7	−0.01	0.01	15/17	−5.4	0.9
Ortega (2004)	P	−3.9	2.31	−20	1.28	−4.61	0.296	1.8	1.11	0.05	0.017		−4.5	0.87
	M	−4.7	2.59	−20.1	1.45	−4.78	0.45	−1.2	1.65	0	0.01		−4.9	0.8

*P/M* pioglitazone group/metformin group

**Table 3:** The difference value between pre and after treatment

Authors	Group	Testosterone		DHEA		FAI		SHBG		TG (mg/dL)		TC (mg/dL)	
		Mean	SD	Mean	SD	Mean	SD	Mean	SD	Mean	SD	Mean	SD
Shahebrahimi (2016)	P	−0.2	0.66	−0.49	1.06					−16.77	49.06	1.28	28.89
	M	−0.2	0.57	−0.26	0.79					−5.07	52.6	2.18	23.47
Chaudhry (2016)	P												
	M												
Sohrevardi (2016)	P			0.2	0.66					−10.7	69.99	−7.9	52.47
	M			−0.1	0.62					6.7	70.86	−5.3	36.85
Kashani (2013)	P	−0.06	1.28	−8.2	96.18					−5.35	57.46	−9.52	33.47
	M	−0.08	1.25	−10.17	94.07					−3.95	59.63	−5.7	32.17
Sangeeta (2012)	P	−0.08	0.55			−5.24	3.64	50.05	14.11			−38.94	10.47
	M	−0.19	0.49			−1.64	3.47	−10.3	13.09			−11.86	13.42
Ziaee (2012)	P									−20.42	42.37	−11.28	31.59
	M									−20.04	48.47	−12.34	37.87
Navali (2012)	P												
	M												
Naka (2011)	P					−4.6	6.63	3.9	18.56	−2	23.52	21	41.8
	M					−5.1	7.69	1.6	16.96	6	38.04	−7	23.26
Cho (2009)	P					−1.4	1.04	9.7	2.77				
	M					−1.8	0.9	3.2	2.91				
Ortega (2005)	P												
	M												
Ortega (2004)	P	−0.95	0.37	−27.4	33.7					−14.9	14.6	−15.1	7.65
	M	−0.86	0.31	−6.2	18.5					−26.8	10.14	−1.8	6.85

*P/M* pioglitazone group/metformin group

### The therapeutic effects of pioglitazone in comparison with metformin

#### Improvement of the menstrual cycle and ovulation

**Fig. 2:**
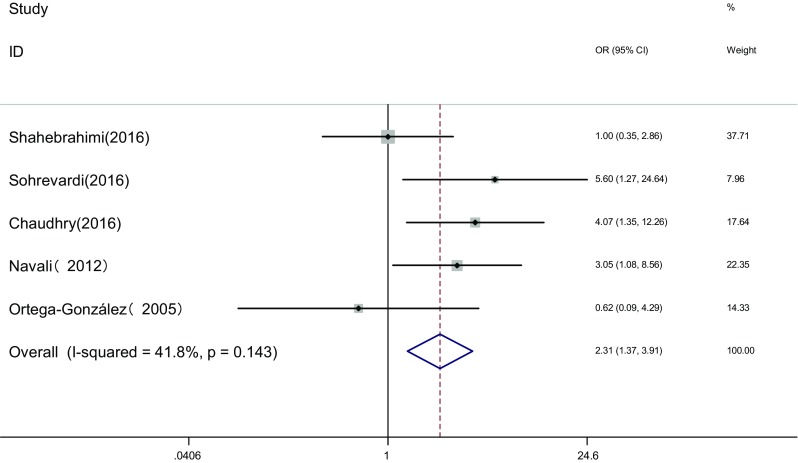
The effect of pioglitazone or metformin on menstrual cycle and ovulation in patients with polycystic ovary syndrome. Overall pioglitazone superior to metformin. *OR* Odds ratio, *CI* confidence interval

There were five articles [16, 20, 24–26] in which menstrual cycle and ovulation were compared between two groups. Heterogeneity test showed no statistical heterogeneity among the studies. (*I* ^2^ = 41.8%, *P* = 0.143). The fixed effects model was adopted for meta-analysis because of the clinical homogeneity. Results showed that difference of total effective rate was statistically significant between the two groups [OR = 2.31, 95% CI (1.37, 3.91), *P* < 0.001]. It showed that in pioglitazone treatment group the curative effect of improving menstrual cycle and ovulation was superior to that in metformin treatment group (Fig. 2).

#### Effect of sex hormones and the clinical manifestations

Only one article compared LH/FSH, hirsutism, acne and hair loss before and after the therapy between the two groups, so the above-mentioned factors were not analyzed.

**Fig. 3:**
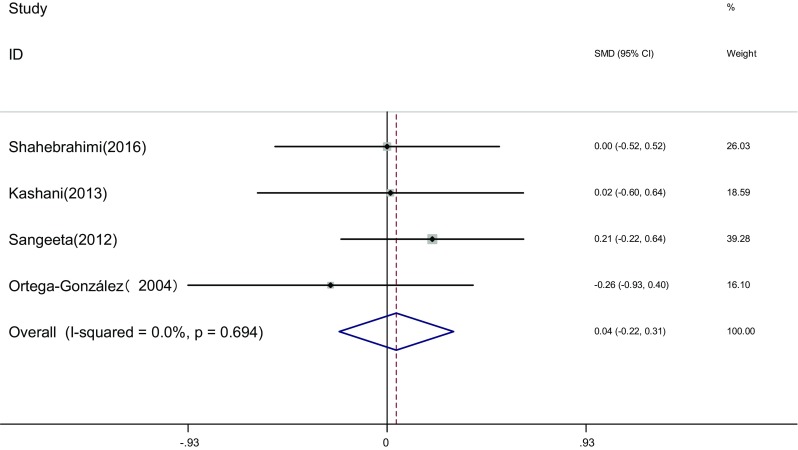
The effect of pioglitazone or metformin on free testosterone in patients with polycystic ovary syndrome. Overall no differences between two therapies. *SMD* standardized mean difference, *CI* confidence interval

Measure of free testosterone: Free testosterone of the two groups was measured before and after the therapy in four articles [17, 21, 23, 25]. Heterogeneity test showed no statistical heterogeneity among the studies (*I* ^2^ = 0.0%, *P* = 0.694). The fixed effects model was adopted for meta-analysis because of the clinical homogeneity. Results showed that difference of free testosterone was not significant between the two groups [SMD = 0.04, 95% CI (−0.22, 0.31), *P* > 0.05]. It showed that there was no difference of free testosterone induced by pioglitazone or metformin (Fig. 3).

**Fig. 4:**
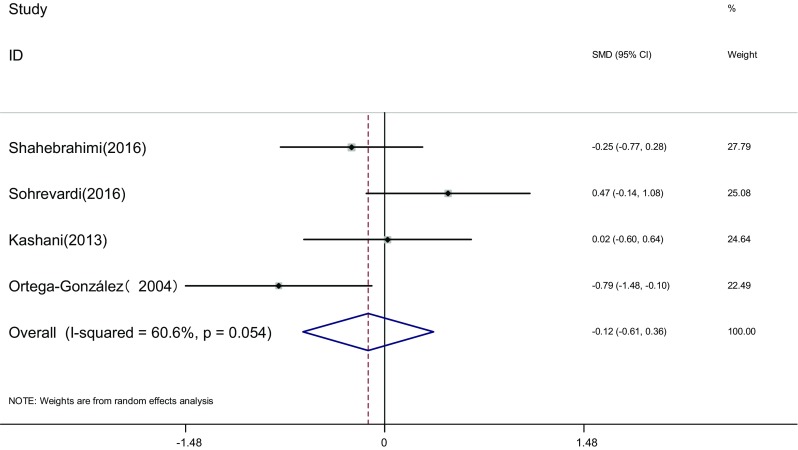
The effect of pioglitazone or metformin on DHEA in patients with polycystic ovary syndrome. Overall no differences between two therapies. *SMD* standardized mean difference, *CI* confidence interval

Measure of DHEA: DHEA of the two groups was measured before and after the therapy in four articles [17, 23, 25, 26]. Heterogeneity test showed significant statistical heterogeneity among the studies (*I* ^2^ = 60.6%, *P* = 0.054). The random effects model was adopted for meta-analysis because of the clinical heterogeneity. Results showed that difference of DHEA was not significant between the two groups [SMD = −0.12, 95% CI (−0.61, 0.36), *P* > 0.05]. It showed that there was no difference of DHEA induced by pioglitazone or metformin (Fig. 4).

**Fig. 5:**
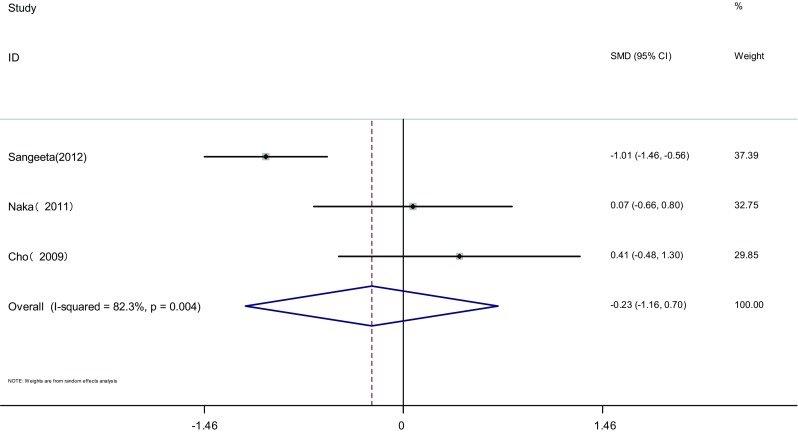
The effect of pioglitazone or metformin on FAI in patients with polycystic ovary syndrome. Overall no differences between two therapies. *SMD* standardized mean difference, *CI* confidence interval

Measure of FAI: FAI of the two groups was measured before and after the therapy in three articles [18, 19, 21]. Heterogeneity test showed significant statistical heterogeneity among the studies (*I* ^2^ = 82.3%, *P* = 0.004). The random effects model was adopted for meta-analysis because of the clinical heterogeneity. Results showed that difference of FAI was not significant between the two groups [SMD = −0.23, 95% CI (−1.16, 0.7), *P* > 0.05]. It showed that there was no difference of FAI induced by pioglitazone or metformin (Fig. 5).

**Fig. 6:**
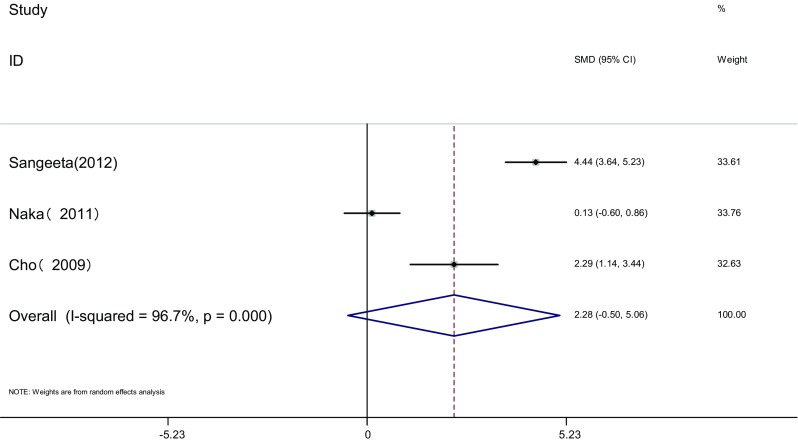
The effect of pioglitazone or metformin on SHBG in patients with polycystic ovary syndrome. Overall no differences between two therapies. *SMD* standardized mean difference, *CI* confidence interval

Measure of SHB: SHBG of the two groups was measured before and after the therapy in three articles [18, 19, 21]. Heterogeneity test showed significant statistical heterogeneity among the studies (*I* ^2^ = 96.7%, *P* < 0.001). The random effects model was adopted for meta-analysis because of the clinical heterogeneity. Results showed that difference of SHBG was not significant between the two groups [SMD = 2.28, 95% CI (−0.5, 5.06), *P* > 0.05]. It showed that there was no difference of SHBG induced by pioglitazone or metformin (Fig. 6).

**Fig. 7:**
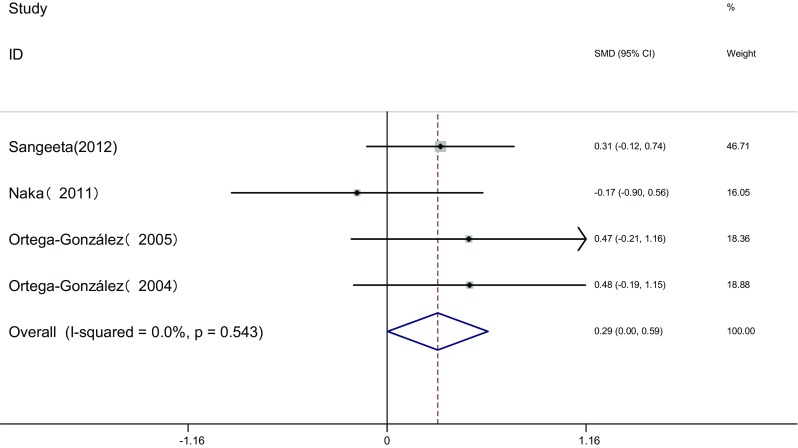
The effect of pioglitazone or metformin on F-G scores in patients with polycystic ovary syndrome. Overall metformin superior to pioglitazone. *SMD* standardized mean difference, *CI* confidence interval

Measure of F-G score: F-G score of the two groups was measured before and after the therapy in four articles [16, 17, 19, 21]. Heterogeneity test showed no statistical heterogeneity among the studies (*I* ^2^ = 0.0%, *P* = 0.543). The fixed effects model was adopted for meta-analysis because of the clinical homogeneity. Results showed that difference of F-G score was significant between the two groups [SMD = 0.29, 95% CI (0.0, 0.59), *P* = 0.048]. It showed that in metformin treatment group the improvement of F-G score was superior to that in pioglitazone treatment group (Fig. 7).

#### Effects on glucose metabolism

**Fig. 8:**
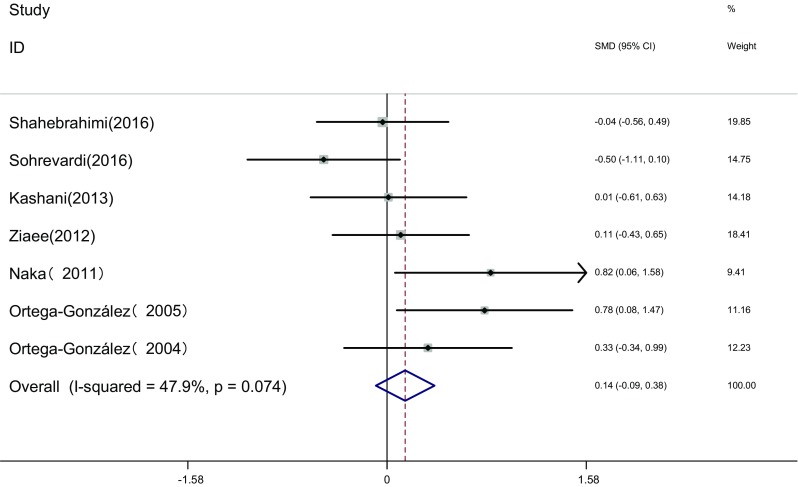
The effect of pioglitazone or metformin on FBS in patients with polycystic ovary syndrome. Overall no differences between two therapies. *SMD* standardized mean difference, *CI* confidence interval

Measure of FBS: FBS of the two groups was measured before and after the therapy in seven articles [16, 17, 19, 22, 23, 25, 26]. Heterogeneity test showed no statistical heterogeneity among the studies (*I* ^2^ = 47.9%, *P* = 0.074). The fixed effects model was adopted for meta-analysis because of the clinical homogeneity. Results showed that the difference of FBS was not significant between the two groups [SMD = 0.14, 95% CI (−0.09, 0.38), *P* > 0.05]. It showed that there was no difference of FBS induced by pioglitazone or metformin (Fig. 8).

**Fig. 9:**
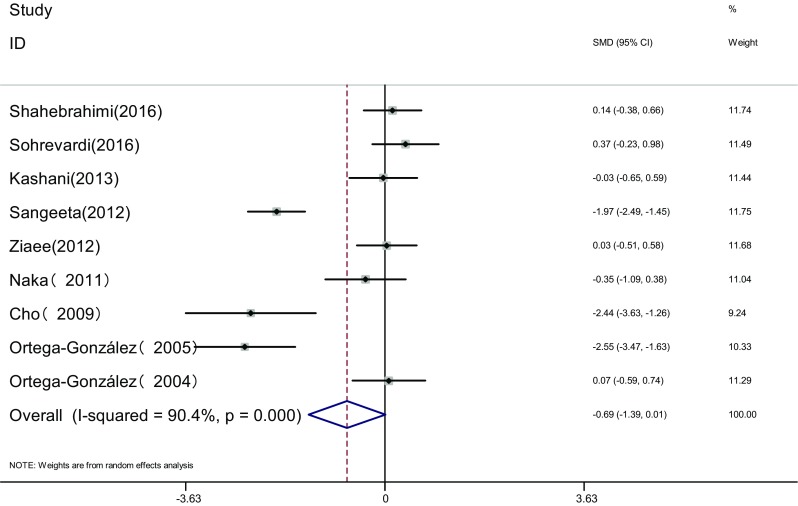
The effect of pioglitazone or metformin on INS in patients with polycystic ovary syndrome. Overall no differences between two therapies. *SMD* standardized mean difference, *CI* confidence interval

Measure of INS: INS of the two groups was measured before and after the therapy in nine articles [16–19, 21–23, 25, 26]. Heterogeneity test showed significant statistical heterogeneity among the studies (*I* ^2^ = 90.4%, *P* < 0.001). The random effects model was adopted for meta-analysis because of the clinical heterogeneity. Results showed that difference of INS was not significant between the two groups [SMD = −0.69, 95% CI (−1.39, 0.01), *P* = 0.054]. It showed that there was no difference of INS induced by pioglitazone or metformin (Fig. 9).

**Fig. 10:**
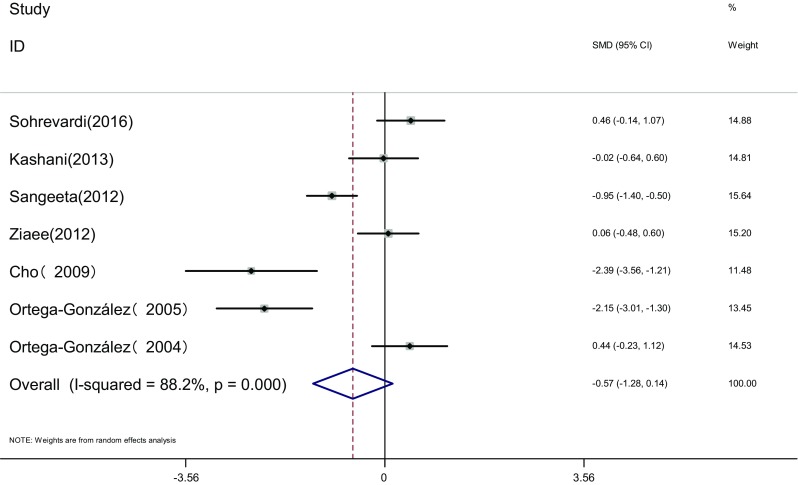
The effect of pioglitazone or metformin on HOMA-IR in patients with polycystic ovary syndrome. Overall no differences between two therapies. *SMD* standardized mean difference, *CI* confidence interval

Measure of HOMA-IR: HOMA-IR of the two groups was measured before and after the therapy in seven articles [16–18, 21–23, 26]. Heterogeneity test showed significant statistical heterogeneity among the studies (*I* ^2^ = 88.2%, *P* < 0.001). The random effects model was adopted for meta-analysis because of the clinical heterogeneity. Results showed that difference of HOMA-IR was not significant between the two groups [SMD = −0.57, 95% CI (−1.28, 0.14), *P* > 0.05]. It showed that there was no difference of HOMA-IR induced by pioglitazone or metformin (Fig. 10).

#### Effects on other metabolic indexes

Only one article compared WC, AST and ALT before the therapy with those after the therapy, so the above-mentioned factors were not analyzed.

**Fig. 11:**
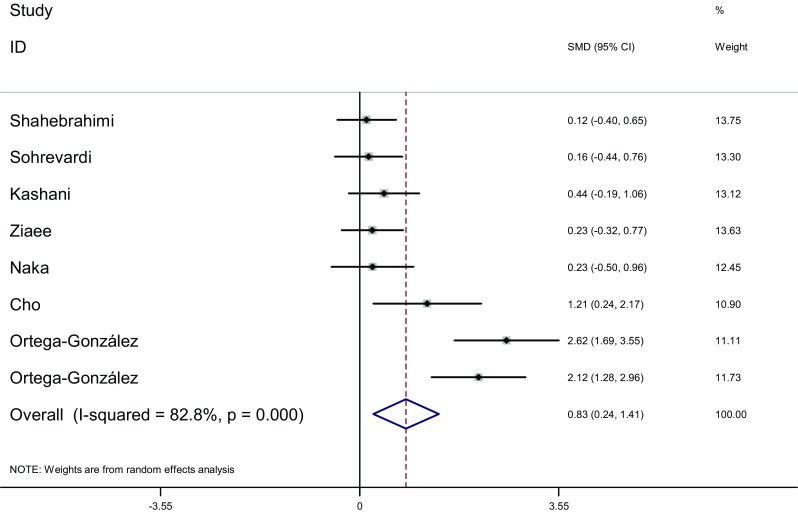
The effect of pioglitazone or metformin on BMI in patients with polycystic ovary syndrome. Overall pioglitazone added more than metformin. *SMD* standardized mean difference, *CI* confidence interval

Measure of BMI: BMI of the two groups was measured before and after the therapy in eight articles [16–19, 22, 23, 25, 26]. Heterogeneity test showed significant statistical heterogeneity among the studies (*I* ^2^ = 82.8%, *P* < 0.001). The random effects model was adopted for meta-analysis because of the clinical heterogeneity. Results showed that difference of BMI was significant between the two groups [SMD = 0.83, 95% CI (0.24, 1.41), *P* = 0.006]. It showed that in pioglitazone treatment group BMI added more than that in metformin treatment group (Fig. 11).

**Fig. 12:**
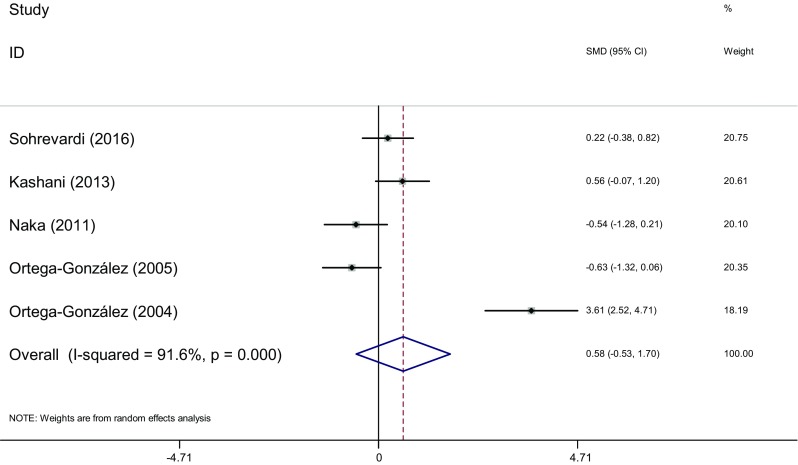
The effect of pioglitazone or metformin on WHR in patients with polycystic ovary syndrome. Overall no differences between two therapies. *SMD* standardized mean difference, *CI* confidence interval

Measure of WHR: WHR of the two groups was measured before and after the therapy in eight articles [16, 17, 20, 23, 26]. Heterogeneity test showed significant statistical heterogeneity among the studies (*I* ^2^ = 91.6%, *P* < 0.001). The random effects model was adopted for meta-analysis because of the clinical heterogeneity. Results showed that difference of WHR was not significant between the two groups [SMD = 0.58, 95% CI (−0.53, 1.7), *P* > 0.05]. It showed that there was no difference of WHR induced by pioglitazone or metformin (Fig. 12).

**Fig. 13:**
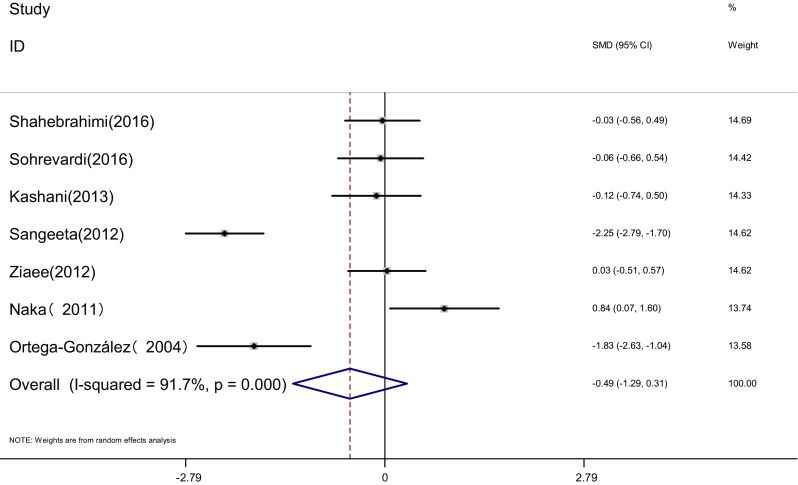
The effect of pioglitazone or metformin on TC in patients with polycystic ovary syndrome. Overall no differences between two therapies. *SMD* standardized mean difference, *CI* confidence interval

Measure of TC: TC of the two groups was measured before and after the therapy in seven articles [17, 19, 21–23, 25, 26]. Heterogeneity test showed significant statistical heterogeneity among the studies (*I* ^2^ = 91.7%, *P* < 0.001). The random effects model was adopted for meta-analysis because of the clinical heterogeneity. Results showed that difference of TC was not significant between the two groups [SMD = −0.49, 95% CI (−1.29, 0.31), *P* > 0.05]. It showed that there was no difference of TC induced by pioglitazone or metformin (Fig. 13).

**Fig. 14:**
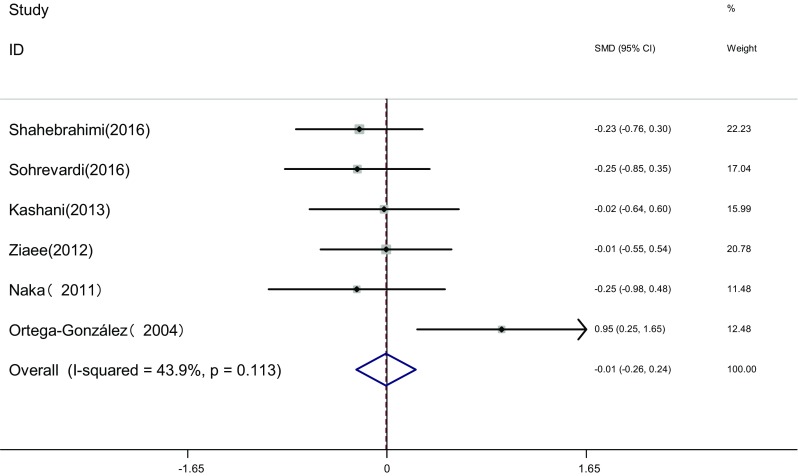
The effect of pioglitazone or metformin on TG in patients with polycystic ovary syndrome. Overall no differences between two therapies. *SMD* standardized mean difference, *CI* confidence interval

Measure of TG: TG of the two groups was measured before and after the therapy in six articles [17, 19, 22, 23, 25, 26]. Heterogeneity test showed no statistical heterogeneity among the studies (*I* ^2^ = 43.9%, *P* = 0.113). The fixed effects model was adopted for meta-analysis because of the clinical homogeneity. Results showed that difference of TG was not significant between the two groups [SMD = −0.01, 95% CI (−0.26, 0.24), *P* > 0.05]. It showed that there was no difference of TG induced by pioglitazone or metformin (Fig. 14).

### Analysis of publication bias

**Fig. 15:**
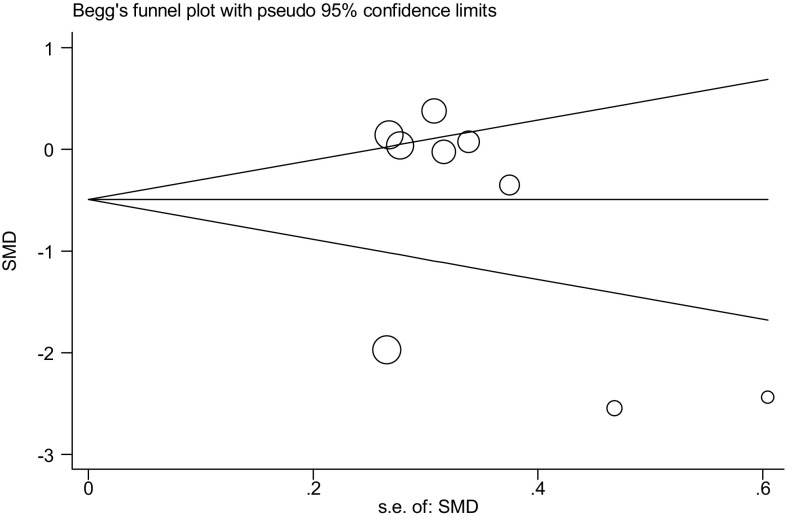
The Begg’s funnel plot of the literatures

The possible publication bias was analyzed by Begg’s and Egger’s test (Table 4, *P* > 0.05). In Begg’s funnel plot (Fig. 15), the researches included were distributed relatively symmetrically. The results showed that there was less likely publication bias.

**Table 4:** Begg’s test and Egger’s test for publication bias

Begg’s test
Adj. Kendall’s score (*P* − *Q*) = −12
Std. dev. of score = 9.59
Number of studies = 9
*Z* = − 1.25
Pr > |*z*| = 0.211
*z* = 1.15 (continuity corrected)
Pr > |*z*| = 0.251 (continuity corrected)

Std_eff	Coeff.	Std. Err.	*t*	*P* > |t|	(95% conf. interval)	
Slope	1.265101	1.523029	0.83	0.434	−2.336289	4.866492
Bias	−5.548647	4.676475	−1.19	0.274	−16.60675	5.509459

### Analysis of meta-regression

Publication year, number of cases, therapeutic doses of pioglitazone and duration of therapy were taken as covariant for meta-regression analysis. It showed that the above factors did not result in the significant heterogeneity of the study (Table 5, *P* > 0.05).

**Table 5:** Meta-regression of publication year, cases, doses and duration

Meta-regression	Number of obs. = 9
REM estimate of between-study variance	*τ* ^2^ = 1.587
% Residual variation due to heterogeneity	*I* ^2^_res = 91.64%
Proportion of between-study variance explained	Adj *R* ^2^ = −30.25%
Joint test for all covariates	Model i(4, 4) = 0.62
With Knapp–Hartung modification	Prob > *F* = 0.6744

ES	Coeff.	Std. Err.	t	P > |t|	(95% conf. interval)	
Year	0.1455123	0.1630263	0.89	0.423	−0.3071213	0.5981459
Cases	−0.0525965	0.055606	−0.95	0.398	−0.2069836	0.1017906
Doses	−1.881142	2.141576	−0.88	0.429	−7.82109	4.064825
Duration	−0.4571394	0.9636579	−0.47	0.660	−3.132683	2.218404
_cons	−286.2016	328.1339	−0.87	0.432	−1197.247	624.8441

### Sensitivity analysis

**Fig. 16:**
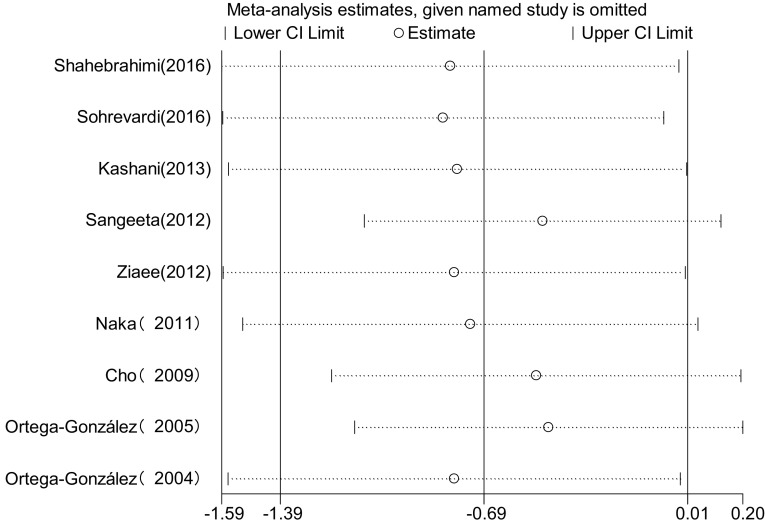
Meta-analysis for sensitivity analysis of the literatures

To investigate how much a single research affecting overall effect size by sensitivity analysis. Results showed that each of the researches included had no significant influence on the overall effect size, i.e., there might be certain heterogeneity among the researches included, but the heterogeneity did not affect the results significantly. So the results of meta-analysis were quite steady (Fig. 16).

## Discussion

Metformin can significantly reduce body mass and fasting insulin levels in patients with PCOS. It can obviously improve insulin sensitivity, hyperandrogenism, menstrual cycle and ovulation. Metformin is relatively safe for the fetus during pregnancy (FDA Pregnancy Category B) [27–29]. Thiazolidinediones (TZDs) are the PPAR-γ (peroxisome proliferator-activated receptor gamma) agonists and act by activating PPARs (nuclear receptors) with greatest specificity. Free fatty acids (FFAs) and eicosanoids are the endogenous ligands for the receptors. When activated, the receptor binds to DNA in complex with the retinoid X receptor (RXR), another nuclear receptor, increasing transcription of a number of specific genes and decreasing transcription of others. That regulated a series of specific gene expression including adiponectin, resistin, leptin, and TNF-α. And that would speed up the differentiation of preadipocytes. Mature adipose cells would be more sensitive to insulin and have the effects of anti-inflammatory. TZDs can decrease the insulin resistance, modify the adipocyte differentiation [30], inhibit the VEGF-induced angiogenesis [31], decrease leptin levels (perhaps leading to an increased appetite) and have the effects of anti-inflammatory. TZDs include rosiglitazone, pioglitazone and troglitazone. Troglitazone has been eliminated due to its serious liver toxicity. In 2010, the FDA carried on the strict restrictions of rosiglitazone for possible increased risk of cardiovascular events. Its restriction was relieved in December 2015 because related researches had not confirmed the increased risk. Animal experiments and epidemiological survey found that pioglitazone might lead to increased risk of bladder cancer. In recent years, many clinical studies have shown that pioglitazone treatment is not associated with increased bladder cancer risk [32–39]. In 2017, FDA determined that [40] the public should be informed about the uncertainty in the literature, while retaining the current warning against the use of pioglitazone in patients with active bladder cancer and for careful considerations of risks and benefits in patients with a prior history of bladder cancer because the results of literatures were inconsistent [32, 41, 42]. A possible association with bladder cancer has also largely been refuted in the consensus statement by the American Association of Clinical Endocrinologists (AACE) and American College of Endocrinology (ACE) on the comprehensive type 2 diabetes management algorithm-2017 executive summary [43]. Pioglitazone was not recommended for PCOS in the clinical practice guideline of AES in 2013 [10]. But TZDs can be used as the second-line choice in the statement of European Society of Endocrinology in 2014 [44]. Our analysis results show that pioglitazone could improve the menstrual cycle and ovulation better than metformin, but significantly increase BMI compared with metformin. Improvement of hirsutism by metformin was superior to that by pioglitazone. Pioglitazone had the similar effects on FBS, INS, HOMA-IR, TG, TC, T, DHEA, FAI, SHBG and WHR as metformin.

The menstrual cycle and ovulation was ameliorated by pioglitazone or metformin in four literatures, and pioglitazone was better than metformin. In nine literatures, pioglitazone obviously elevated BMI but metformin decreased BMI, and this was in accordance with the guideline or consensus. In five literatures, pioglitazone or metformin had no effects on WHR; there was no difference between the two agents and this indirectly showed that pioglitazone elevated BMI not for increasing central obesity. Studies in diabetics suggest that pioglitazone can increase peripheral fat and may reduce visceral fat stores [45]. Pioglitazone can reduce ectopic fat including the liver and muscle, and thus enhance tissue sensitivity to insulin [46, 47]. In some patients, TZDs may increase body weight due to fluid retention [44]. In four literatures, F-G scores of hirsutism were decreased by pioglitazone or metformin, and metformin was a little better than pioglitazone. In seven literatures, FBS, insulin and HOMA-IR were deceased by pioglitazone or metformin, and there were no differences between the two agents. The effects on androgen and lipid were different in the literatures. Many literatures showed that pioglitazone ameliorated androgen and lipid better than metformin, but there was no difference between the two agents from meta-analysis. Based on different dosages and time of treatment, it shows that the pioglitazone therapy for 3 or 6 months seems better than that for 1.5 months, and either 30 or 45 mg of pioglitazone therapy is effective. But 45 mg of pioglitazone therapy seems more BMI gained. Side effects may be mitigated using a moderate dose (e.g., ≤30 mg) of pioglitazone [43]. So 30 mg daily of pioglitazone therapy for 3–6 months may be a better choice for PCOS. There was only descriptive information and few statistical data of side effects in most literatures. Adverse reactions such as headache, rash and myalgia were minor. Side effects were recorded and compared in detail only in one literature of Kashani. Gastrointestinal reaction was more significant in metformin group than in pioglitazone group. Pioglitazone increased appetite more than metformin. The two agents both had no effects on liver function such as AST and ALT.

There are some deficiencies in this study because apparent heterogeneity exists in some data of different literatures. We analyzed the possible causes of the heterogeneity: (1) there are not enough literatures conformed to the diagnostic criteria and inclusion criteria entering our study. The number of patients in each research is from 20 to 50, so there are not very enough cases in the literatures. Multi-center large RCT studies were insufficient and there might be some sampling error. (2) There were no detailed instructions for the race of patients in the researches. Only one study gave clear indication for the race of the Caucasus ethnic groups, and others did not; (3) not all the research methods of the literatures are perfect. Blind method was conducted only in two articles. Concealment and analysis of intention-to-treat (ITT) was conducted only in one article. There were no specific statistics on the data of the patients retreated or loss to follow-up. There was no analysis if the patients retreated for pregnancy might benefit from the medicine which could lead to conception or if that might decrease the efficiency of the agents. All of the above might increase the error of the results. (4) The dosages of both pioglitazone and metformin used in the individual studies are very heterogeneous. Dosages of pioglitazone were 30 and 45 mg per day, and dosages of metformin were 1000, 1500, 1700, and 2550 mg per day. This fact might strongly affect the outcomes of the studies. (5) The duration of the individual studies is likewise very heterogeneous—some were 3 and others 6 months long and one just 1.5 months. This might also affect the outcomes of the studies. The course of treatment was short which was from 6 weeks to 6 months. This might lead to inconsistent results which were dependent on time. (6) No exact statistics were for side effects. Most only described as symptoms such as rash, nausea, and vomiting. Only in one article were there detailed data such as liver function before and after treatment in both groups.

## Conclusions

Our study shows that pioglitazone improved menstrual cycle and ovulation of PCOS patients better than metformin. In contrast with pioglitazone, metformin could reduce weight and ameliorate symptoms of hirsutism better. Two agents had similar effects on other metabolic targets. Pioglitazone may be an alternative treatment in insulin-resistant or obese PCOS women who do not tolerate or do not respond to metformin therapy and 30 mg daily of pioglitazone therapy for 3–6 months may be a better choice. In the future, high quality and multi-center study of RCT with sufficient cases are required to confirm the curative effects and side effects of pioglitazone.
